# Fuzzy Approach Analyzing SEIR-SEI Dengue Dynamics

**DOI:** 10.1155/2020/1508613

**Published:** 2020-10-14

**Authors:** G. Bhuju, G. R. Phaijoo, D. B. Gurung

**Affiliations:** ^1^Department of Mathematics, Bhaktapur Multiple Campus, Bhaktapur, Nepal; ^2^Department of Mathematics, School of Science, Kathmandu University, Dhulikhel, Nepal

## Abstract

Dengue fever is a mosquito-borne infectious disease threatening more than a hundred tropical countries of the world. The heterogeneity of mosquito bites of human during the spread of dengue virus is an important factor that should be considered while modeling the dynamics of the disease. However, traditional models assumed homogeneous transmission between host and vectors which is inconsistent with reality. Mathematically, we can describe the heterogeneity and uncertainty of the transmission of the disease by introducing fuzzy theory. In the present work, we study transmission dynamics of dengue with the fuzzy SEIR-SEI compartmental model. The transmission rate and recovery rate of the disease are considered as fuzzy numbers. The dynamical behavior of the system is discussed with different amounts of dengue viruses. Also, the fuzzy basic reproduction number for a group of infected individuals with different virus loads is calculated using Sugeno integral. Simulations are made to illustrate the mathematical results graphically.

## 1. Introduction

Dengue is one of the major public health concerns. It is a mosquito-borne fastest growing tropical disease in the world. Dengue cases have been increasing from 2.2 million (in 2010) to over 3.2 million (in 2015) across the America, Southeast Asia, and Western Pacific. More than one-third of the world's population, approximately 3.9 billion people, are living in the dengue risk area of 128 countries [1]. Annually, about 390 million new dengue infections are occurring worldwide [2]. There is no licensed vaccine and no specific antiviral drugs for the disease. About 2.5% of those that are infected by dengue die [1].

Dengue is one of the emerging diseases in Nepal. The outbreak of dengue usually occurs in Nepal during June to October. Dengue case was first reported in Nepal 2004, and major outbreaks have occurred on 2006, 2010, 2013, 2016, and 2019 [3]. In the year 2019, more than 14,662 DENV infection cases were confirmed in 67 districts of Nepal. Among them, six people were reported to die due to dengue disease infection [4].

Dengue fever is caused by one of the four closely related dengue viruses of different serotypes: DENV-1, DENV-2, DENV-3, DENV-4, which circulate simultaneously in an endemic area. Dengue viruses are single-stranded RNA viruses of the Flaviviridae family. It is transmitted by the day-feeding mosquito Aedes Aegypti and the Asian Tiger mosquito, Aedes Albopictus [5].

Mathematical models are more effective tools to understand transmission dynamics of the disease, to identify the influential parameters in spreading the disease, and to propose strategies for the control of the disease. There is long and distinguished history of using mathematical models for the study of the evolution and transmission dynamics of infectious diseases. Kermack and Mckendrick formulated an SIR compartmental model to study infectious diseases mathematically [6]. Esteva and Vargas remodeled it to use for vector host dynamics of dengue disease taking constant [7] and variable human population [8]. Since then, many researchers have studied dengue disease transmission dynamics. Gakkhar and Chavda and Phaijoo and Gurung studied the impact of awareness on the spread of dengue infection in a human population [9, 10]. Mobility of human population causes the spread of the disease in new human populations, so impact of these mobility parameters has been studied through mathematical models [11].

In the modeling of the transmission of dengue disease, several nonlinear models of ordinary differential equations have been used [6, 7, 10–12]. In these models, the variables commonly represent subpopulations of susceptible (*S*), exposed (*E*), infectious (*I*), and recovered (*R*).

Most of the researchers have used deterministic models with constant model parameters. Generally, they assumed that each individual can transmit the disease and recover from the disease in a constant rate. But these assumptions conflicted with real epidemic. The model parameters like transmission rates, biting rates, and recovery rates are uncertain. Zadeh [13] had introduced the uncertainty in a biological model. To study this uncertainty, mathematically, he defined the fuzzy set and fuzzy theory. Mondal et al. modified the epidemic SIS model by considering the disease transmission parameter and treatment control parameter as fuzzy number [14]. De Barros et al. applied fuzzy theory technique on a SI epidemiological model while considering different degrees of infectivity. Also, they used the transmission coefficient as a fuzzy set [15]. Djam and Wajiga presented a fuzzy expert system for management of malaria, to provide the decision support platform to malaria researchers [16]. Emokhare and Igbape proposed a fuzzy logic-based approach for the early diagnosis of Ebola hemorrhagic fever [17].

Recently, fuzzy theory has been introduced in many models of engineering, banking, public health, and biology. Also, the theory has been used to study the diagnosis of the diseases. Previous studies regarding the general epidemic models (SI, SIR) of infectious disease have been developed with a fuzzy transmission parameter for the diseases which are transmitted to human from human directly [14, 15, 18]. Dengue is an infectious disease which cannot be transmitted to human from human directly without the intermediate vector, the Aedes mosquito. In the disease dynamics, the transmission rate and recovery rate of the disease are not deterministic; they are uncertain. So, in the present work, we consider these parameters as fuzzy numbers. We compute fuzzy basic reproduction number to study the stability of the equilibrium points.

This paper is organized as follows. Fuzzy set and fuzzy expected value are defined in Subsections 1.1 and 1.2. In Section 2, we present the analysis of the fuzzy epidemiological model. Also, positivity and boundedness of the solution of the model are described in Subsection 2.2. In Section 3, we perform stability analysis with basic reproduction number of the dengue disease, using a next-generation matrix method. We present a fuzzy basic reproduction number and compare it with the deterministic basic reproduction number with different virus loads of dengue disease in Subsection 3.3. In Section 4, numerical results and discussion about the work are presented.

### 1.1. Fuzzy Set

Let *X* be a nonempty crisp set. A fuzzy subset *S* of *X* is denoted by $\tilde{S}$ and is defined as

(1) $$\begin{array}{c} \tilde{S}=\left\{\left(x,\mu_{\tilde{S}}\left(x\right)\right)\text{: }x\in X\right\}, \end{array}$$

where $\mu_{\tilde{S}}:X⟶\left[0,1\right]$ is a membership function associated with a fuzzy set $\tilde{S}$ which describes the degree of belongingness of *x* with *X*.

Here, we use the membership function *μ*(*x*) to indicate the fuzzy subset $\tilde{S}$. Also, *μ*(*x*) is called fuzzy number if *X* is the set of real numbers.

### 1.2. Fuzzy Measure and Fuzzy Expected Value

Let *Ω* be a nonempty set and *P*(*Ω*) denote the set of all subsets of *Ω*. Then, *μ* : *P*(*Ω*)⟶[0, 1] is a fuzzy measure [19] if

- *μ*(*ϕ*) = 0 and *μ*(*Ω*) = 1
- for *A*, *B* ∈ *P*(*Ω*), *μ*(*A*) ≤ *μ*(*B*) if *A* ⊂ *B*

Let *u* : *Ω*⟶[0, 1] be an uncertain variable; i.e., *u* is a fuzzy subset and *μ* a fuzzy measure on *Ω*. Then, fuzzy expected value (FEV) of *u* is the real number, defined by the Sugeno integral [19],

(2) $$\begin{array}{c} \text{FEV}\left(u\right)=\int_{\Omega }u\;d\mu =\sup\left\{\inf\left(\alpha ,k\left(\alpha \right)\right)\right\},\;0\leq \alpha \leq 1, \end{array}$$

where

(3) $$\begin{array}{c} k\left(\alpha \right)=\mu \left\{\omega \in \Omega :u\left(\omega \right)\geq \alpha \right\}. \end{array}$$

## 2. Fuzzy SEIR-SEI Model of Dengue

In this paper, we propose a SEIR-SEI model for dengue transmission by incorporating the fuzzy number. The model describes the interaction between susceptible, exposed, infected, and recovered human population and susceptible, exposed, and infected mosquito population by the system of nonlinear ordinary differential equations [11]. In the deterministic model proposed in [11], we use the fuzzy number. Among the individuals of the population, there are different degrees of susceptibility and infectivity, so the concept of susceptible and infectious is uncertain. Focusing on the population heterogeneity, we consider the disease transmission coefficient *β*_*h*_ between susceptible and infected individuals as a fuzzy number. The recovery of the infection of the disease is also uncertain. The infected individual will recover from the disease, when the amount of virus is reducing from the body. So, we consider that the recovery rate *γ*_*h*_ is also a fuzzy number. To describe the virus load on these parameters, we use the membership function *β*_*h*_(*v*) and *γ*_*h*_(*v*) for the transmission rate and recovery rate, respectively. Then, the fuzzy SEIR-SEI model of dengue disease is described by the following system of differential equations:

(4) $$\begin{array}{c} \frac{dS_{h}}{dt}=\mu_{h}N_{h}-\frac{\beta_{h}\left(v\right)b}{N_{h}}S_{h}I_{m}-\mu_{h}S_{h},\\ \frac{dE_{h}}{dt}=\frac{\beta_{h}\left(v\right)b}{N_{h}}S_{h}I_{m}-\left(k_{h}+\mu_{h}\right)E_{h},\\ \frac{dI_{h}}{dt}=k_{h}E_{h}-\left(\gamma_{h}\left(v\right)+\mu_{h}\right)I_{h},\\ \frac{dR_{h}}{dt}=\gamma_{h}\left(v\right)I_{h}-\mu_{h}R_{h},\\ \frac{dS_{m}}{dt}=A-\frac{\beta_{m}b}{N_{h}}S_{m}I_{h}-\mu_{m}S_{m},\\ \frac{dE_{m}}{dt}=\frac{\beta_{m}b}{N_{h}}S_{m}I_{h}-\left(k_{m}+\mu_{m}\right)E_{m},\\ \frac{dI_{m}}{dt}=k_{m}E_{m}-\mu_{m}I_{m}. \end{array}$$

Here, *N*_*h*_ = *S*_*h*_ + *E*_*h*_ + *I*_*h*_ + *R*_*h*_ and *N*_*m*_ = *S*_*m*_ + *E*_*m*_ + *I*_*m*_, where *N*_*h*_ is the host (human) population size, *S*_*h*_ is number of susceptibles in the host population, *I*_*h*_ is the number of infectives in the host population, *R*_*h*_ number of immunes (recovered) in the host population, *N*_*m*_ is the vector (mosquito) population size, *S*_*m*_ is the number of susceptibles in the vector population, *I*_*m*_ is the number of infectives in the vector population, *μ*_*h*_ is the birth/death rate in the host population, *μ*_*m*_ is the death rate in the vector population, *β*_*h*_ is the transmission coefficient from vector to host, *β*_*m*_ is the transmission coefficient from host to vector, *γ*_*h*_ is the recovery rate in the host population, *b* is the biting rate of vector, *k*_*h*_ is the host's incubation rate, and *k*_*m*_ is the vector's incubation rate.

### 2.1. Membership Function

The fuzzy membership function of the transmission parameter *β*_*h*_(*v*) which depends on the amount of virus load *v* is given by [15, 20]

(5) $$\begin{array}{c} \beta_{h}\left(v\right)=\left\{\begin{array}{cc} 0, & \text{if}\;v<v_{\min},\\ \frac{v-v_{\min}}{v_{M}-v_{\min}}, & \text{if}\;v_{\min}\leq v\leq v_{M},\\ 1 & \text{if}\;v_{M}\leq v\leq v_{\max} \end{array}\right. \end{array}$$

**Figure 1:**
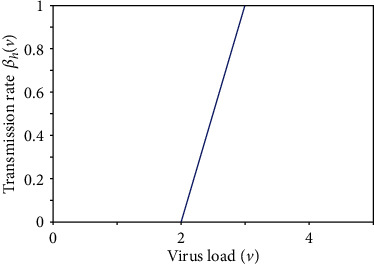
Membership function *β*_*h*_(*v*).

Here, *v*_min_ represents the minimum amount of virus needed for the disease transmission to occur. When the amount of virus in an individual is less than *v*_min_, the chance of transmission of disease is negligible. Moreover, for the certain amount of virus *v*_*M*_, the transmission rate of the disease is maximum and equal to 1. Furthermore, we suppose that for the dengue disease, the individual's amount of virus is always limited by *v*_max_. The diagram of *β*_*h*_(*v*) is given in Figure 1.

**Figure 2:**
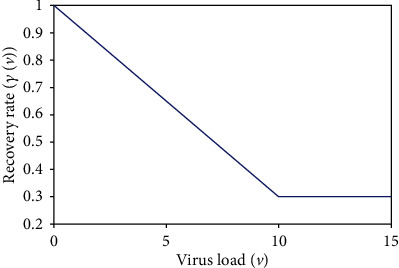
Membership function *γ*_*h*_(*v*).

Here, *γ*_*h*_(*v*) represents the recovery rate from the infection of the disease which depends on the amount of virus load. When the virus load is higher, it will take a longer time to recovery from the disease. Thus, the fuzzy membership function of recovery rate *γ*_*h*_(*v*) is given by [18]

(6) $$\begin{array}{c} \gamma_{h}\left(v\right)=\frac{\left(\gamma_{0}-1\right)}{v_{\max}}v+1,\;\text{if}\;0<v<v_{\max}, \end{array}$$

where 0 < *γ*_0_ < 1 is the lowest recovery rate. The diagram of *γ*_*h*_(*v*) is given in Figure 2.

We assume that the amount of virus of the studied group *V* may be different for different individuals. So, with the classification of the studied group given by an expert, it can be seen as a linguistic variable such as weak, medium, and strong. Each classification of the linguistic variable with membership function Γ(*v*) is given by [15]

(7) $$\begin{array}{c} \Gamma \left(v\right)=\left\{\begin{array}{c} 0,\;\text{if}\;v<\bar{v}-\delta ,\\ \frac{v-\bar{v}+\delta }{\delta },\;\text{if}\;\bar{v}-\delta \leq v\leq \bar{v},\\ \frac{-\left(v-\bar{v}-\delta \right)}{\delta },\;\text{if}\;\bar{v}<v\leq \bar{v}+\delta ,\\ 1,\;\text{if}\;v>\bar{v}+\delta . \end{array}\right. \end{array}$$

**Figure 3:**
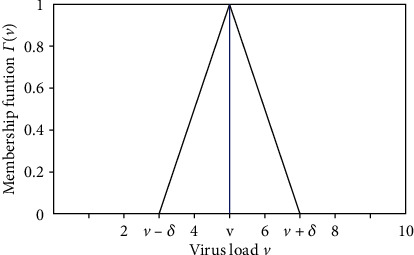
Membership function Γ(*v*).

The parameter $\bar{v}$ represents a central value, and *δ* is the dispersion of each one of the fuzzy set assumed by *V*. The diagram of Γ(*v*) is given in Figure 3.

### 2.2. Nonnegativity and Boundedness

**Theorem 1 .**

The solutions of the system (4) are nonnegative for all *t* > 0.

**Proof**

Suppose *D* = {(*S*_*h*_, *E*_*h*_, *I*_*h*_, *R*_*h*_, *S*_*m*_, *E*_*m*_, *I*_*m*_) ∈ ℝ^7^ : 0 ≤ *S*_*h*_, *E*_*h*_, *I*_*h*_, *R*_*h*_, *S*_*m*_, *E*_*m*_, *I*_*m*_}.

We show that *D* should be positively invariant. To prove it, we examine the behavior of the state variables at the boundaries of *D*.

- At the boundary *S*_*h*_ = 0, we get

(8) $$\begin{array}{c} S_{h}^{\prime }=\mu_{h}N_{h}>0 \end{array}$$

Thus, the solution cannot exit *D* by crossing this boundary.

(b) At the boundary *E*_*h*_ = 0, we get,

(9) $$\begin{array}{c} E_{h}^{\prime }=\frac{\beta_{h}\left(v\right)b}{N_{h}}S_{h}I_{m} \end{array}$$

**Case 1 .**

If *E*_*h*_ = 0, *S*_*h*_ > 0, and *I*_*m*_(*t*) > 0, then *E*_*h*_′ > 0.

**Case 2 .**

If *E*_*h*_ = 0, *S*_*h*_ > 0, and *I*_*m*_ = 0, then *E*_*h*_′ = 0.

**Case 3 .**

If *E*_*h*_ = 0, *S*_*h*_ = 0, and *I*_*m*_ > 0, then *E*_*h*_′ = 0.

In each of these cases, *E*_*h*_′ ≥ 0, so the solution cannot exit *D*, by crossing the boundary *E*_*h*_ = 0.

(c) At the boundary *I*_*h*_ = 0, we have *I*_*h*_′ = *k*_*h*_*E*_*h*_

If *I*_*h*_ = 0, *E*_*h*_ > 0, then *I*_*h*_′ > 0.

Thus, the solution cannot exit *D*, by crossing the boundary *I*_*h*_ = 0.

In the similar manner, we can show that the solution of the system cannot exit *D* by crossing the boundary of any of the state variables.

**Theorem 2 .**

The solutions of the system (4) are bounded on [0, *b*) for some *b* > 0.

**Proof**

We have from the system (4) *N*_*h*_ = *S*_*h*_ + *E*_*h*_ + *I*_*h*_ + *R*_*h*_ and *dN*_*h*_/*dt* = 0. Thus, *N*_*h*_ is constant for all *t* ∈ [0, *b*) for some *b* > 0. Therefore, *S*_*h*_(*t*), *E*_*h*_(*t*), *I*_*h*_(*t*), *R*_*h*_(*t*) are all bounded on [0, *b*).

Again, we have,

(10) $$\begin{array}{c} N_{m}=S_{m}+E_{m}+I_{m}, \end{array}$$

which implies

(11) $$\begin{array}{c} \frac{dN_{m}}{dt}=A-\mu_{m}N_{m},\\ N_{m}=\frac{A}{\mu_{m}}+\left(N_{m}\left(0\right)-\frac{A}{\mu_{m}}\right)e^{-\mu_{m}t}. \end{array}$$

Hence,

(12) $$\begin{array}{c} \underset{t\to \infty }{\lim\sup}N_{m}\leq \frac{A}{\mu_{m}}. \end{array}$$

Therefore, *S*_*m*_(*t*), *E*_*m*_(*t*), and *I*_*m*_(*t*) are bounded above by *A*/*μ*_*m*_ on [0, *b*) for some *b* > 0. Since all the variables are nonnegative, these are bounded below by 0. Hence, the solution of the system (4) are bounded on [0, *b*) for some *b* > 0 [21].

### 2.3. Existence and Uniqueness

Here, we show the existence and uniqueness of solutions of the model (4). We assume that the system has the initial conditions as follows:

(13) $$\begin{array}{c} S_{h}\left(0\right)>0,\\ E_{h}\left(0\right)\geq 0,\\ I_{h}\left(0\right)>0,\\ R_{h}\left(0\right)\geq 0,\\ S_{m}\left(0\right)>0,\\ E_{m}\left(0\right)\geq 0,\\ I_{m}\left(0\right)\geq 0. \end{array}$$

**Theorem 3 .**

Consider the system (4) with nonnegative initial condition (13). Solutions to the system (4) with initial conditions (13) exist and are unique for all *t* ≥ 0.

**Proof**

Let *x*(*t*) = (*S*_*h*_(t), *E*_*h*_(*t*), *I*_*h*_(*t*), *R*_*h*_(*t*), *S*_*m*_(*t*), *E*_*m*_(*t*), *I*_*m*_(*t*)) ∈ ℝ^7^. The system (4) is written in the form *x*′ = *f*(*x*). Let *f*_*i*_, *i* = 1, 2, 3, 4, 5, 6, 7 denote the components of the vector field f; we have

(14) $$\begin{array}{c} f_{1}=\mu_{h}N_{h}-\frac{\beta_{h}\left(v\right)b}{N_{h}}S_{h}I_{m}-\mu_{h}S_{h},\\ f_{2}=\frac{\beta_{h}\left(v\right)b}{N_{h}}S_{h}I_{m}-\left(k_{h}+\mu_{h}\right)E_{h},\\ f_{3}=k_{h}E_{h}-\left(\gamma_{h}\left(v\right)+\mu_{h}\right)I_{h},\\ f_{4}=\gamma_{h}\left(v\right)I_{h}-\mu_{h}R_{h},\\ f_{5}=A-\frac{\beta_{m}b}{N_{h}}S_{m}I_{h}-\mu_{m}S_{m},\\ f_{6}=\frac{\beta_{m}b}{N_{h}}S_{m}I_{h}-\left(k_{m}+\mu_{m}\right)E_{m},\\ f_{7}=k_{m}E_{m}-\mu_{m}I_{m}. \end{array}$$

The vector field *f* consists of the algebraic polynomials of state variables. Thus, *f*_*i*_ are continuous autonomous functions on ℝ^7^ and partial derivatives *∂f*_*i*_/*∂S*_*h*_, *∂f*_*i*_/*∂E*_*h*_, *∂f*_*i*_/*∂I*_*h*_, *∂f*_*i*_/*∂R*_*h*_, *∂f*_*i*_/*∂S*_*m*_, *∂f*_*i*_/*∂E*_*m*_, and *∂f*_*i*_/*∂I*_*m*_ exist and are continuous. Hence, by existence and uniqueness theorem, a unique solution of the system *x*′ = *f*(*x*) exists for any initial condition *x*(0) ∈ ℝ^7^ [22].

## 3. Stability Analysis of the Model

### 3.1. Basic Reproduction Number

Basic reproduction number is defined as the average number of secondary infections caused by a single infectious individual during their entire infectious lifetime [23, 24]. The number is denoted by *R*_0_.

Assume that *F* is the matrix of transmission terms and *V* is the matrix of transition terms of the system (4). *R*_0_ is defined as the spectral radius of the matrix *FV*^−1^, i.e., *ρ*(*FV*^−1^). *R*_0_ is obtained by using the next-generation matrix method [23, 24]. For the model (4),

(15) $$\begin{array}{c} F=\left[\begin{array}{cccc} 0 & 0 & 0 & \frac{\beta_{h}\left(v\right)b}{N_{h}}S_{h}\\ 0 & 0 & \frac{\beta_{m}b}{N_{h}}S_{m} & 0\\ 0 & 0 & 0 & 0\\ 0 & 0 & 0 & 0 \end{array}\right],\\ V=\left[\begin{array}{cccc} p & 0 & 0 & 0\\ 0 & \alpha & 0 & 0\\ -k_{h} & 0 & q & 0\\ 0 & -k_{m} & 0 & \mu_{m} \end{array}\right],\\ p=k_{h}+\mu_{h},\;\alpha =k_{m}+\mu_{m},\text{and }q=\gamma_{h}\left(v\right)+\mu_{h}. \end{array}$$

Thus, the basic reproduction number is

(16) $$\begin{array}{c} R_{0}\left(v\right)=\rho \left(FV^{-1}\right)=\sqrt{\frac{\beta_{h}\left(v\right)\beta_{m}b^{2}k_{m}k_{h}A}{\mu_{m}^{2}\alpha pqN_{h}}.} \end{array}$$

### 3.2. Equilibrium Points

There are two equilibrium points of the system of differential equations (4), the disease-free equilibrium point *P*_0_(*N*_*h*_, 0, 0, 0, *A*/*μ*_*m*_, 0, 0) and endemic equilibrium point *P*_1_(*S*_*h*_^∗^, *E*_*h*_^∗^, *I*_*h*_^∗^, *R*_*h*_^∗^, *S*_*m*_^∗^, *E*_*m*_^∗^, *I*_*m*_^∗^). Here,

(17) $$\begin{array}{c} S_{h}^{*}=\frac{N_{h}\left[R_{0}^{2}\mu_{h}\mu_{m}N_{h}\alpha +\beta_{h}\left(v\right)bAk_{m}\right]}{R_{0}^{2}\left(\mu_{h}\mu_{m}N_{h}\alpha +\beta_{h}\left(v\right)bAk_{m}\right)},\\ E_{h}^{*}=\frac{\left(R_{0}^{2}-1\right)\mu_{h}\mu_{m}^{2}N_{h}^{2}q\alpha }{\beta_{m}bk_{h}\left(\beta_{h}\left(v\right)bAk_{m}+\mu_{h}\mu_{m}N_{h}\alpha \right)},\\ I_{h}^{*}=\frac{\left(R_{0}^{2}-1\right)\mu_{h}\mu_{m}^{2}N_{h}^{2}\alpha }{\left(\beta_{h}\left(v\right)b^{2}A\beta_{m}k_{m}+\mu_{h}\mu_{m}N_{h}\alpha \beta_{m}b\right)},\\ R_{h}^{*}=\frac{\left(R_{0}^{2}-1\right)\gamma_{h}\left(v\right)\mu_{m}^{2}N_{h}^{2}\alpha }{\beta_{m}b\left(\beta_{h}\left(v\right)bAk_{m}+\mu_{h}\mu_{m}N_{h}\alpha \right)},\\ S_{m}^{*}=\frac{A\left(\beta_{h}\left(\text{v}\right)bAk_{m}+\mu_{h}\mu_{m}N_{h}\alpha \right)}{R_{0}^{2}\mu_{h}\mu_{m}^{2}N_{h}\alpha +\mu_{m}\beta_{h}\left(v\right)bAk_{m}},\\ E_{m}^{*}=\frac{\left(R_{0}^{2}-1\right)\mu_{h}N_{h}A\mu_{m}}{\left[R_{0}^{2}\mu_{h}\mu_{m}N_{h}\alpha +\beta_{h}\left(v\right)bAk_{m}\right]},\\ I_{m}^{*}=\frac{\left(R_{0}^{2}-1\right)\mu_{h}N_{h}Ak_{m}}{\left[R_{0}^{2}\mu_{h}\mu_{m}N_{h}\alpha +\beta_{h}\left(v\right)bAk_{m}\right]}. \end{array}$$

**Theorem 4 .**

The disease-free equilibrium point *P*_0_(*N*_*h*_, 0, 0, 0, *A*/*μ*_*m*_, 0, 0) is locally asymptotically stable when *R*_0_ < 1 and unstable when *R*_0_ > 1.

**Proof**

The Jacobian matrix of system of Equation (4) is

(18) $$\begin{array}{c} J=\left[\begin{array}{ccccccc} -\frac{\beta_{h}\left(v\right)b}{N_{h}}I_{m}-\mu_{h} & 0 & 0 & 0 & 0 & 0 & -\frac{\beta_{h}\left(v\right)b}{N_{h}}S_{h}\\ \frac{\beta_{h}\left(v\right)b}{N_{h}}I_{m} & -p & 0 & 0 & 0 & 0 & \frac{\beta_{h}\left(v\right)b}{N_{h}}S_{h}\\ 0 & k_{h} & -q & 0 & 0 & 0 & 0\\ 0 & 0 & \gamma_{h}\left(v\right) & -\mu_{h} & 0 & 0 & 0\\ 0 & 0 & -\frac{\beta_{m}b}{N_{h}}S_{m} & 0 & \frac{\beta_{m}b}{N_{h}}I_{h}-\mu_{m} & 0 & 0\\ 0 & 0 & \frac{\beta_{m}b}{N_{h}}S_{m} & 0 & \frac{\beta_{m}b}{N_{h}}I_{h} & -\alpha & 0\\ 0 & 0 & 0 & 0 & 0 & k_{m} & -\mu_{m} \end{array}\right]. \end{array}$$

The characteristic equation at the disease-free equilibrium point is

(19) $$\begin{array}{c} \;|\;J-\lambda I\;|\;=0\Rightarrow {\left(\mu_{h}+\lambda \right)}^{2}\left(\mu_{m}+\lambda \right)\left[-\left(p+\lambda \right)\left(q+\lambda \right)\left(\alpha +\lambda \right)\left(\mu_{m}+\lambda \right)+\frac{\beta_{m}b^{2}\beta_{h}\left(v\right)k_{h}k_{m}A}{N_{h}\mu_{m}}\right]=0. \end{array}$$

Therefore,

(20) $$\begin{array}{c} \lambda =-\mu_{h},-\mu_{h},-\mu_{m},\\ \lambda^{4}+A_{1}\lambda^{3}+A_{2}\lambda^{2}+A_{3}\lambda +A_{4}=0, \end{array}$$

where

(21) $$\begin{array}{c} A_{1}=p+q+\alpha +\mu_{m},\\ A_{2}=p\;q+\alpha \mu_{m}+\left(p+q\right)\left(\alpha +\mu_{m}\right),\\ A_{3}=p\;q\left(\alpha +\mu_{m}\right)+\alpha \;\mu_{m}\left(p+q\right),\\ A_{4}=p\;q\;\alpha \;\mu_{m}\left(1-R_{0}^{2}\right). \end{array}$$

*β*_*h*_(*v*) and *γ*_*h*_(*v*) have different values for different virus loads. Thus, we have the following three cases for virus loads (*v*).

**Case 4 .**

*v* < *v*_min_,

(22) $$\begin{array}{c} \beta_{h}\left(v\right)=0\Rightarrow R_{0}^{2}=0,\\ \gamma_{h}\left(v\right)=\left(\gamma_{0}-1\right)/v_{\max}v+1>0. \end{array}$$

Since the parameters *p*, *q*, *α*, and *μ*_*m*_ are all positive, in the above condition,

(23) $$\begin{array}{c} A_{1}>0,\;A_{3}>0,\;A_{4}>0,\\ A_{1}A_{2}-A_{3}=\left(p+q+\alpha +\mu_{m}\right)\left[pq+\alpha \mu_{m}+\left(p+q\right)\left(\alpha +\mu_{m}\right)\right]-\left[pq\left(\alpha +\mu_{m}\right)+\alpha \mu_{m}\left(p+q\right)\right]=\left(p+q\right)pq+\alpha \mu_{m}\left(\alpha +\mu_{m}\right)+\left(p+q+\alpha +\mu_{m}\right)\left(p+q\right)\left(\alpha +\mu_{m}\right)>0,\\ A_{1}A_{2}A_{3}=\left(p+q+\alpha +\mu_{m}\right)\left[pq+\alpha \mu_{m}+\left(p+q\right)\left(\alpha +\mu_{m}\right)\right]\left[pq\left(\alpha +\mu_{m}\right)+\alpha \mu_{m}\left(p+q\right)\right]=p^{2}q^{2}\left(p+q\right)\left(\alpha +\mu_{m}\right)+p^{2}q^{2}{\left(\alpha +\mu_{m}\right)}^{2}+2pq\alpha \mu_{m}\left(p+q\right)\left(\alpha +\mu_{m}\right)+pq\alpha \mu_{m}{\left(\alpha +\mu_{m}\right)}^{2}+pq{\left(p+q\right)}^{2}{\left(\alpha +\mu_{m}\right)}^{2}+pq\left(p+q\right){\left(\alpha +\mu_{m}\right)}^{3}+pq\alpha \mu_{m}{\left(p+q\right)}^{2}+\alpha^{2}\mu_{m}^{2}{\left(p+q\right)}^{2}+\alpha^{2}\mu_{m}^{2}\left(p+q\right)\left(\alpha +\mu_{m}\right)+\alpha \mu_{m}{\left(p+q\right)}^{3}\left(\alpha +\mu_{m}\right)+\alpha \mu_{m}{\left(p+q\right)}^{2}{\left(\alpha +\mu_{m}\right)}^{2},\\ A_{3}^{2}=p^{2}q^{2}{\left(\alpha +\mu_{m}\right)}^{2}+\alpha^{2}\mu_{m}^{2}{\left(p+q\right)}^{2}+2pq\alpha \mu_{m}\left(p+q\right)\left(\alpha +\mu_{m}\right),\\ A_{4}A_{1}^{2}=pq\alpha \mu_{m}\left[{\left(p+q\right)}^{2}+{\left(\alpha +\mu_{m}\right)}^{2}+2\left(p+q\right)\left(\alpha +\mu_{m}\right)\right],\\ A_{1}A_{2}A_{3}-A_{3}^{2}-A_{4}A_{1}^{2}=\left(p+q\right)\left(\alpha +\mu_{m}\right)\left[{\left(pq-\alpha \mu_{m}\right)}^{2}+pq\left(p+q\right)\left(\alpha +\mu_{m}\right)+pq{\left(\alpha +\mu_{m}\right)}^{2}\right]+\left(p+q\right)\left(\alpha +\mu_{m}\right)\left[\alpha \mu_{m}{\left(p+q\right)}^{2}+\alpha \mu_{m}\left(p+q\right)\left(\alpha +\mu_{m}\right)\right]>0. \end{array}$$

Therefore, *A*_1_ > 0, *A*_4_ > 0, *A*_3_ > 0, *A*_1_*A*_2_ − *A*_3_ > 0, and *A*_1_*A*_2_*A*_3_ − *A*_3_^2^ − *A*_4_*A*_1_^2^ > 0.

**Case 5 .**

*v*_min_ ≤ *v* ≤ *v*_*M*_,

(24) $$\begin{array}{c} \beta_{h}\left(v\right)=\frac{v-v_{\min}}{v_{M}-v_{\min}},\\ \gamma_{h}\left(v\right)=\frac{\left(\gamma_{0}-1\right)}{v_{\max}}v+1>0. \end{array}$$

We have *A*_1_ > 0, *A*_3_ > 0, *A*_1_*A*_2_ − *A*_3_ > 0, *A*_4_ > 0 if 1 − *R*_0_^2^ > 0, *R*_0_^2^ < 1, and *R*_0_ < 1 and *A*_1_*A*_2_*A*_3_ − *A*_3_^2^ − *A*_4_*A*_1_^2^ > 0 if 1 − *R*_0_^2^ > 0, *R*_0_^2^ < 1, and *R*_0_ < 1.

**Case 6 .**

*v*_*M*_ ≤ *v* ≤ *v*_max_

(25) $$\begin{array}{c} \beta_{h}\left(v\right)=1,\\ \gamma \left(v\right)=\frac{\left(\gamma_{0}-1\right)}{v_{\max}}v+1>0. \end{array}$$

We have *A*_1_ > 0, *A*_1_*A*_2_ − *A*_3_ > 0, *A*_3_ > 0, and *A*_4_ ≥ 0 and *A*_1_*A*_2_*A*_3_ − *A*_3_^2^ − *A*_4_*A*_3_^2^ = *A*_1_*A*_2_*A*_3_ − *A*_3_^2^ > 0.

According to the Routh-Hurwitz criterion [25], the roots of Equation (20) have negative roots or roots with negative real parts. Hence, the disease-free equilibrium point is asymptotically stable in all cases.

**Theorem 5 .**

The system has an endemic equilibrium point *P*_1_(*S*_*h*_^∗^, *E*_*h*_^∗^, *I*_*h*_^∗^, *R*_*h*_^∗^, *S*_*m*_^∗^, *E*_*m*_^∗^, *I*_*m*_^∗^) which exists only when *R*_0_ > 1.

**Proof**

We have endemic equilibrium point *P*_1_(*S*_*h*_^∗^, *E*_*h*_^∗^, *I*_*h*_^∗^, *R*_*h*_^∗^, *S*_*m*_^∗^, *E*_*m*_^∗^, *I*_*m*_^∗^), where

(26) $$\begin{array}{c} I_{h}^{*}=\frac{\left(R_{0}^{2}-1\right)\mu_{h}\mu_{m}^{2}N_{h}^{2}\alpha }{\left(\beta_{h}\left(v\right)b^{2}A\beta_{m}k_{m}+\mu_{h}\mu_{m}N_{h}\alpha \beta_{m}b\right)}. \end{array}$$

- When *v* < *v*_min_, we have

(27) $$\begin{array}{c} \beta_{h}\left(v\right)=0\;R_{0}^{2}=0 \end{array}$$

We get

(28) $$\begin{array}{c} I_{h}^{*}=\frac{-\mu_{m}N_{h}}{\beta_{m}b}<0. \end{array}$$

So, the disease dies out.

(b) When *v*_min_ ≤ *v* ≤ *v*_*M*_, we have

(29) $$\begin{array}{c} \beta_{h}\left(v\right)=\frac{v-v_{\min}}{v_{M}-v_{\min}}>0,\\ R_{0}^{2}=\frac{\left(v-v_{\min}\right)\beta_{m}k_{\text{m}}k_{h}b^{2}A}{\left(v_{M}-v_{\min}\right)pq\alpha \mu_{m}^{2}N_{h}}>0. \end{array}$$

So, *I*_*h*_^∗^ > 0 if *R*_0_ > 1. Thus, the disease is endemic.

(c) When *v*_*M*_ ≤ *v* ≤ *v*_max_, we have

(30) $$\begin{array}{c} \beta_{h}\left(v\right)=1,\\ R_{0}^{2}=\frac{\beta_{m}k_{m}k_{h}b^{2}A}{pq\alpha \mu_{m}^{2}N_{h}}>0. \end{array}$$

So, *I*_*h*_^∗^ > 0 if *R*_0_ > 1. Thus, the disease is endemic.

Hence, the endemic equilibrium point exists if virus load *v* ≥ *v*_min_.

### 3.3. Fuzzy Basic Reproduction Number

Since the transmission rate of disease and recovery rate of infection of disease are assumed as a function of the virus load, the basic reproduction number will be a function of the virus load. The classical basic reproduction number *R*_0_(*v*) is increasing with virus load *v*. It is not a fuzzy set, and it can be greater than 1, but *γ*_0_ is always a positive fraction with the highest value 1, so 0 ≤ *γ*_0_*R*_0_(*v*) ≤ 1. Thus, *γ*_0_*R*_0_(*v*) is a fuzzy set and hence, FEV(*γ*_0_*R*_0_(*v*)) is well defined. Under this view, we introduce the fuzzy basic reproduction number of the fuzzy SEIR-SEI model [18].

The fuzzy basic reproduction number is given by

(31) $$\begin{array}{c} R_{0}^{f}=\frac{1}{\gamma_{0}}\text{FEV}\left(\gamma_{0}R_{0}\left(v\right)\right), \end{array}$$

where

(32) $$\begin{array}{c} \text{FEV}\left(\gamma_{0}R_{0}\left(v\right)\right)=\sup\left\{\inf\left(\alpha ,k\left(\alpha \right)\right)\right\},\;0\leq \alpha \leq 1,\\ k\left(\alpha \right)=\mu \left\{v:\gamma_{0}R_{0}\left(v\right)\geq \alpha \right\}=\mu \left(X\right), \end{array}$$

which is a fuzzy measure.

We have to define fuzzy measure *μ* to obtain FEV(*γ*_0_*R*_0_(*v*)). For this, the possibility measure [15]

(33) $$\begin{array}{c} \mu \left(X\right)=\sup\Gamma \left(v\right),\;\forall v\in X,X\subset R. \end{array}$$

We know that *R*_0_(*v*) is not decreasing with *v*; from FEV(*γ*_0_*R*_0_(*v*)), we have $X=\left[\bar{v},v_{\max}\right]$, where $\bar{v}$ is the solution of the equation

(34) $$\begin{array}{c} \gamma_{0}\frac{\beta_{h}\left(v\right)\beta_{m}b^{2}k_{m}k_{h}A}{\mu_{m}^{2}\alpha pqN_{h}}=\alpha . \end{array}$$

Thus, *k*(*α*) = *μ*[*v*′, *v*_max_] = supΓ(*v*) with *v*′ ≤ *v* ≤ *v*_max_, where *k*(0) = 1 and *k*(1) = Γ(*v*_max_).

**Figure 4:**
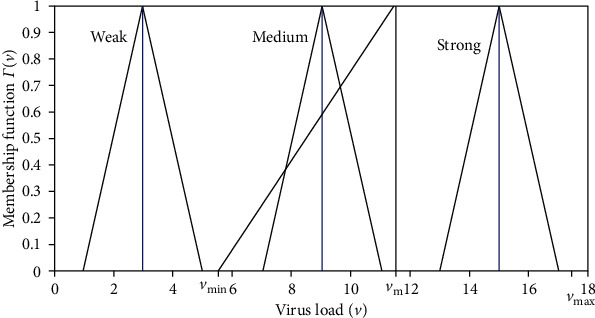
Weak, medium, and strong virus load with transmission rate *β*_*h*_(*v*).

The amount of virus *v* in the population was assumed as a linguistic meaning which is classified into three states: weak virus load, medium virus load, and strong virus load. Each state has a fuzzy behavior based on values *v*_min_, *v*_*M*_, and *v*_max_ described in Figure 4.

**Case 7 .**

Weak virus load when $\bar{v}+\delta \leq v_{\min}$; we have

(35) $$\begin{array}{c} \beta_{h}\left(v\right)=0,\\ R_{0}\left(v\right)=0. \end{array}$$

So, FEV(*γ*_0_*R*_0_(*v*)) = 0 < *γ*_0_⇔*R*_0_^*f*^ < 1.

Then, the disease will die out.

**Case 8 .**

Medium virus load when $\bar{v}-\delta \geq v_{\min}$ and $\bar{v}+\delta \leq v_{M}$; we have

(36) $$\begin{array}{c} \beta_{h}\left(v\right)=\frac{v-v_{\min}}{v_{M}-v_{\min}},\\ R_{0}\left.\left(v\right)\right)=\frac{\beta_{h}\left(v\right)\beta_{m}b^{2}k_{\text{m}}k_{h}A}{\mu_{m}^{2}\alpha pq\left(v\right)N_{h}},\\ k\left(\alpha \right)=\left\{\begin{array}{cc} 1 & \text{if}\;0<\alpha \leq \gamma_{0}R_{0}\left(\bar{v}\right),\\ \Gamma \left(v^{\prime }\right), & \text{if }\gamma_{0}R_{0}\left(\bar{v}\right)<\alpha \leq \gamma_{0}R_{0}\left(\bar{v}+\delta \right)\\ 0 & \text{if }\gamma_{0}R_{0}\left(\bar{v}+\delta \right)<\alpha \leq 1 \end{array}\right. \end{array}$$

Thus, if *δ* > 0, *k*(*α*) is continuous and a decreasing function with *k*(0) = 1 and *k*(1) = 0. Hence, the FEV(*γ*_0_*R*_0_(*v*)) is the fixed point of *k* and

(37) $$\begin{array}{c} \gamma_{0}R_{0}\left(\bar{v}\right)\leq \text{FEV}\left(\gamma_{0}R_{0}\left(v\right)\right)\leq \gamma_{0}R_{0}\left(\bar{v}+\delta \right),\\ R_{0}\left(\bar{v}\right)\leq R_{0}^{f}\leq R_{0}\left(\bar{v}+\delta \right). \end{array}$$

As a function $R_{0}\left(\bar{v}\right)$ is increasing and a continuous function, by intermediate value theorem, there exist $\overset{˘}{v}$ with $\bar{v}<\overset{˘}{v}<\bar{v}+\delta$ such that

(38) $$\begin{array}{c} R_{0}^{f}=R_{0}\left(\overset{˘}{v}\right)>R_{0}\left(\bar{v}\right). \end{array}$$

Thus, there exists a virus load $\overset{˘}{v}$ such that $R_{0}^{f}=R_{0}\left(\overset{˘}{v}\right)$; i.e., *R*_0_^*f*^ and $R_{0}\left(\overset{˘}{v}\right)$ coincide. Furthermore, $R_{0}^{f}>R_{0}\left(\bar{v}\right)$. Therefore, the fuzzy average number of secondary infection *R*_0_^*f*^ is higher than the number of secondary infection $R_{0}\left(\bar{v}\right)$ due to the medium amount of virus.

**Case 9 .**

Strong virus load when $\bar{v}-\delta \leq v_{M}$ and $\bar{v}+\delta \leq v_{\max}$; we have

(39) $$\begin{array}{c} \beta_{h}\left(v\right)=1,\\ R_{0}\left(v\right)=\frac{\beta_{m}b^{2}k_{m}k_{h}A}{\mu_{m}^{2}\alpha pq\left(\bar{v}\right)N_{h}},\\ k\left(\alpha \right)=\left\{\begin{array}{c} 1,\;\text{if}\;0<\alpha \leq \gamma_{0}R_{0}\left(\bar{v}\right),\\ \Gamma \left(v^{\prime }\right),\;\text{if}\;\gamma_{0}R_{0}\left(\bar{v}\right)<\alpha \leq \gamma_{0}R_{0}\left(\bar{v}+\delta \right),\\ 0,\;\text{if}\;\gamma_{0}R_{0}\left(\bar{v}+\delta \right)<\alpha \leq 1. \end{array}\right. \end{array}$$

As in Case 2, we have

(40) $$\begin{array}{c} \gamma_{0}R_{0}\left(\bar{v}\right)\leq \text{FEV}\left(\gamma_{0}R_{0}\left(v\right)\right)\leq \gamma_{0}R_{0}\left(\bar{v}+\delta \right),\\ \frac{\beta_{m}b^{2}k_{m}k_{h}A}{\mu_{m}^{2}\alpha pq\left(\bar{v}\right)N_{h}}\leq \frac{1}{\gamma_{0}}\text{FEV}\left(\gamma_{0}R_{0}\left(v\right)\right)\leq \frac{\beta_{m}b^{2}k_{m}k_{h}A}{\mu_{m}^{2}\alpha pq\left(\bar{v}+\delta \right)N_{h}},\\ R_{0}\left(\bar{v}\right)\leq R_{0}^{f}\leq R_{0}\left(\bar{v}+\delta \right). \end{array}$$

Thus, *R*_0_^*f*^ > 1; the disease will be endemic.

## 4. Numerical Results and Discussion

We explore the influence of dengue virus load with the fuzzy behavior on the transmission dynamics of the dengue disease. We have simulated the fuzzy SEIR-SEI model with different values of dengue virus loads, 357, 5 × 10^7^, and 1.04 × 10^10^ RNA per ml [26, 27].

**Figure 5:**
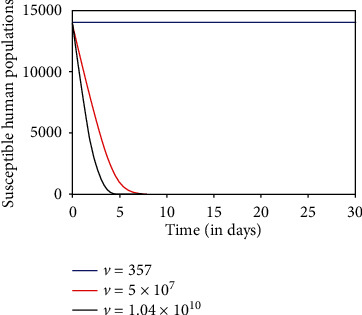
Susceptible human population.

**Figure 6:**
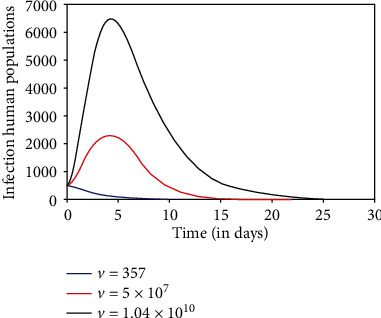
Infectious human population.

**Figure 7:**
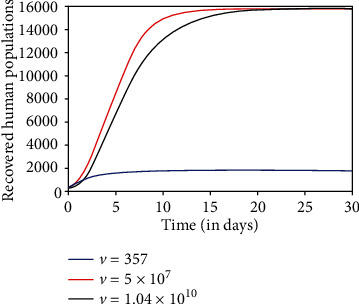
Recovered human population.

As virus load *v* increases, the infection rate increases. It causes the increase in an infectious human population. So, the susceptible human population decreases. Figures 5–7 describe the dynamics of susceptible, infectious, and recovered host, respectively, for different virus loads. When the virus load is minimum (357 RNA per ml), the infectivity of the disease is negligible, so there is no transmission of the disease at this moment of virus load (Figure 5). It is noted that when the virus load is maximum (1.04 × 10^10^ RNA per ml), the susceptible human population decreases significantly to its least value (Figure 5), due to significant increases in transmission rate. Initially, the infectious human population increases due to high transmission rate. Later on, the population starts decreasing due to recovery from the disease and natural death (Figures 6 and 7).

To illustrate the dynamics of dengue disease transmission with the fuzzy behavior, different values of parameters are needed (Table 1).

**Figure 8:**
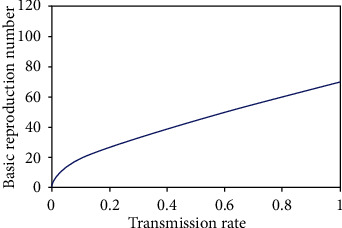
Basic reproduction number with transmission rate of disease.

**Figure 9:**
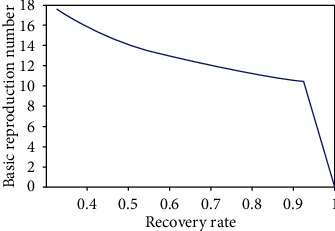
Basic reproduction number with recovery rate of disease.

**Figure 10:**
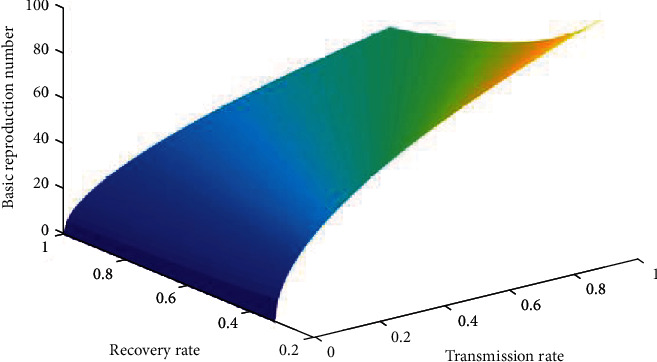
Basic reproduction number with transmission rate and recovery rate of disease.

Basic reproduction number indicates whether the dengue disease will vanish or persists in the population over time. When the fuzzy transmission rate of the disease increases, the infectious population increases, so the basic reproduction number also increases (Figure 8). The basic reproduction number decreases with the increase of the recovery rate of the disease. From maximum to the medium virus loads (10^7^ RNA per ml), the basic reproduction number decreases slightly, since at that situation, infection of the disease will be very high. After that, it decreases very sharply (Figure 9); because the recovery rate is very high, it means the virus load is very low. Thus, when transmission rate increases, the disease will be endemic and when recovery rate increases, the disease will die out (Figure 10).

Different phenomena of transmission dynamics of the dengue disease are observed due to fuzziness of the model parameters which are considered functions of virus loads. These phenomena cannot be observed in deterministic models. Thus, the fuzzy model can describe transmission dynamics of dengue in a more realistic than deterministic model of the disease.

## 5. Conclusion

In this paper, we have studied the SEIR-SEI epidemic model of dengue disease in a crisp and fuzzy system. We have studied the dynamical behavior of the system. We considered the two parameters, transmission rate of disease and recovery rate, from infection as a function of virus loads and defined their fuzzy membership functions. Also, we analyzed the stability of the model at disease-free equilibrium point with different virus loads. We computed the fuzzy basic reproduction number.

Dengue disease cannot be spread among the population if the amount of dengue virus is very low due to the natural immunity. It will be endemic if the amount of the virus is high. In the fuzzy system, these phenomena could be considered, but it cannot be considered in the crisp system. In the classical system, the basic reproduction number is the function of system parameters only, whereas in the fuzzy system, the basic reproduction number is a function of disease-spreading virus. Thus, the fuzzy model is more realistic, flexible, and balanced than the crisp model of the dengue disease.

The uncertain model parameters, transmission rate and recovery rate, play a crucial role in the disease transmission dynamics. So, we have considered only these parameters as a function of dengue virus loads. We may consider the other uncertain parameters of the model as the function of virus.

## Figures and Tables

**Table 1:** Parameters and their values.

Parameters	*μ*_*h*_	*k*_*h*_	*N*_*h*_	*μ*_*m*_	*k*_*m*_	*b*	*β*_*m*_	*A*
Values	1/(70∗365)	0.5	160000	0.02941	0.1428	0.75	0.375	250000
Units	Day ^−1^	Day ^−1^	Number	Day ^−1^	Day ^−1^	Day ^−1^	Dimensionless	Number/day

## Data Availability

The data used to support the findings of this study are included within the article.
